# Emergency Logistics in a Large-Scale Disaster Context: Achievements and Challenges

**DOI:** 10.3390/ijerph16050779

**Published:** 2019-03-04

**Authors:** Yiping Jiang, Yufei Yuan

**Affiliations:** 1 Department of Management Engineering, Nanjing Agricultural University, Nanjing 210031, China; 2 DeGroote School of Business, McMaster University, Hamilton, ON M8S 4M4, Canada; yuanyuf@mcmaster.ca

**Keywords:** large-scale disaster, emergency response, emergency logistics, humanitarian logistics, operations research

## Abstract

There is growing research interest in emergency logistics within the operations research (OR) community. Different from normal business operations, emergency response for large scale disasters is very complex and there are many challenges to deal with. Research on emergency logistics is still in its infancy stage. Understanding the challenges and new research directions is very important. In this paper, we present a literature review of emergency logistics in the context of large-scale disasters. The main contributions of our study include three aspects: First, we identify key characteristics of large-scale disasters and assess their challenges to emergency logistics. Second, we analyze and summarize the current literature on how to deal with these challenges. Finally, we discuss existing gaps in the relevant research and suggest future research directions.

## 1. Introduction

Large scale disasters, such as Haiti’s earthquake in January 2010 and Japan’s biggest earthquake, tsunami and nuclear reactor meltdown in March 2011, often happen suddenly and cause large casualties and significant damages to society. In general, a disaster can be defined as “a shocking event that seriously disrupt the functioning of a community or society, by causing human, material, economic or environmental damage that cannot be handled by local agencies through standard procedures” [1]. When a large-scale disaster happens, immediate emergency responses are needed in order to save lives and relieve and control the damages [2]. As pointed out by Altay and Green III [3], “Disasters are large intractable problems that test the ability of communities and nations to effectively protect their populations and infrastructure, to reduce both human and property loss, and to rapidly recover. The seeming randomness of impacts and problems and uniqueness of incidents demand dynamic, real-time, effective and cost efficient solutions, thus making the topic very suitable for OR/MS research.” Emergency logistics is “the support function that ensures the timely delivery of emergency resources and rescue services into the affected regions so as to assist in rescue activities [4]” while humanitarian logistics is more focusing on aiding people in their survival during and after a disaster. However, in the research arena, the differences between emergency logistics and humanitarian logistics have been slight [5]. In this work, we will use the term emergency logistics as a general term, and do not emphasize the differences between them.

Many scholars in the OR community have studied emergency logistics, especially after the 2001 9/11 terrorist attack in the U.S. [6,7]. We were able to find six survey papers on emergency logistics that are related to our study. Green and Kolesar [8] analyzed previous OR papers focused on urban emergency services and regular emergencies, published in the INFORMS journals from 1960 to 2004. Wright et al. [9] extended the literature scope into homeland security such as traffic and cyber space safety. Both of these studies focused on OR in routine emergency management, but not in the context of large-scale disasters. Altay and Green III [3] summarized the works of the OR community published from 1980 to 2006 under a broad umbrella of disaster operation management (DOM) in large-scale disasters. They provided a comprehensive literature classification in six dimensions: authors’ affiliation, disaster type, solution methodology, operational stage, research contribution type, and a discipline classification of management science, management engineering, and management consulting based on the extension of Denizel et al. For further research, they highlighted the gaps in multi-agency coordination, soft OR, the use of sensing technology, recovery planning, business continuity, and management engineering. Simpson and Hancock [10] reviewed the operational research (OR) foundation in emergency response from 1965 to 2007. They discussed the previous literature on both urban emergency service systems studied in earlier periods and of large-scale disasters in the 21st century, and they also identified a detailed literature citation network among those studies. Four major areas of opportunity were suggested: soft versus hard OR, information and DSSs, volunteers and temporary organizational structures, and performance metrics in the context of emergency response. Caunhye et al. [11] reviewed optimization models in emergency logistics in the pre-disaster operations phase and post-disaster operations phase. Using content analysis approach, they analyzed the current literature in detail through the perspectives of OR models, decisions, objectives, and constraints. They also suggested some future research problems such as post-disaster dynamic inventory modeling, combining aspects of transportation time, injury seriousness, on-field treatment, and medical center service load for casualty transportation, adding objective measures for coordination effectiveness and proper organizational structure, and taking human behavior uncertainty in disaster into consideration. Most recently, Galindo and Batta [1] reviewed the progress and research gap of OR/MS research in DOM after Altay and Green’s [3] review in 2006. They followed the same review framework of Altay and Green [3] but added an analysis about the most common assumptions in the field. They classified these assumptions into three categories: reasonable, limited, and unrealistic, and emphasized the importance of assumption validation. Finally, they suggested further research directions including: (i) improvement of the coordination among DOM actors; (ii) introduction of new technologies through more application studies; (iii) study of DOM problems using formal statistical analysis to establish realistic assumptions in DOM models; (iv) in-depth exploration of methodologies such as Soft OR and interdisciplinary techniques; and (v) measurement of the effectiveness of adopted strategies. The review papers and their contributions are summarized in Table 1.

Generally, all of the survey papers have summarized and classified the existing literature in emergency logistics. Although they suggested further research areas in general, there is lack of detailed analysis on the gaps between what we have studied and what we should study in emergency logistics that directly address the challenges faced in real world disaster situations.

Our research aims to identify the current research gaps in emergency logistics research in the context of large-scale disasters. Since emergency logistics is significantly different and facing more challenges than traditional business logistics, we take problem identification and solution approach rather than summery and classification approach to our literature review with the following steps. First, we identify the key characteristics of large-scale disasters and corresponding challenges posed to emergency logistics. Second, we analyze current OR efforts on how to deal with these challenges. Finally, we investigate the gaps in current research, and suggest future research directions.

## 2. Survey Scope and Method

Emergency management activities are commonly described in four programmatic phases: mitigation, preparedness, response, and recovery [12]. “Mitigation is the application of measures that will either prevent the onset of a disaster or reduce the impacts should one occur. Preparedness activities prepare the community to respond when a disaster occurs. Response is the employment of resources and emergency procedures as guided by plans to preserve life, property, the environment, and the social, economic, and political structure of the community. Recovery involves the actions taken in the long term after the immediate impact of the disaster has passed to stabilize the community and to restore some semblance of normalcy” [3]. In our study we only concentrate on those OR literatures that investigate emergency logistics in the immediate response phrase and in the context of large-scale disasters.

We searched for literature on emergency logistics published in academic peer-reviewed journals and book chapters, while conference proceedings or working papers are not included. We search the title, abstract and keyword of journal articles published in English only. The search keywords we used contain “disaster”, “large-scale disaster”, “catastrophe”, “emergency”, “emergency response”, “emergency logistics”, “humanitarian logistics”, “optimization”, as well as their combination and extensions such as “disastrous”, “catastrophic”, etc. As the result, we have identified 81 papers on emergency logistics from 42 journals, including *Operations Research*, *Management Science*, *Transportation Research Part E*, *European Journal of Operational Research*, Interfaces, *Journal of the Operational Research Society*, *Interfaces*, *Computers & Operations Research*, *IIE Transactions*, *Annals of Operations Research*, *OR Spectrum*, *Naval Research Logistics*, etc. 

## 3. Key Characteristics of Large-Scale Disasters and Challenges of Emergency Logistics

In contrast with routine emergencies such as medical emergency or traffic accidents, large-scale disasters can result in severe impacts on large concentrations of people, activity, and wealth and last for a longer time. Some specific characteristics of large-scale disasters differ, depending on the type of disaster and the types of relief actors involved, and they pose certain challenges for emergency logistics in the aftermath of disasters [2]. In this section we first identify the common characteristics of large-scale disasters, and then investigate some potential challenges of practical emergency responses.

### 3.1. Key Characteristics of Large-Scale Disasters

As pointed out by Chen et al. [13], a typical emergency situation has characteristics such as, “great uncertainty; sudden and unexpected events; the risk of possible mass casualty; high amounts of time pressure and urgency; severe resource shortages; large-scale impact and damage; and the disruption of infrastructure support necessary for coordination like electricity, telecommunications, and transportation. This is complicated by factors such as infrastructure interdependencies; multi-authority and massive personal involvement; conflict of interest; and the high demand for timely information.” Recently, Holguin-Veras et al. [14] also analyzed the unique features of post-disaster humanitarian logistics. They further distinguished the term catastrophes from disaster where catastrophes were defined as “high-consequence events that generate widespread and crippling impacts, where the ability of the impacted society to respond is severely compromised”. It seems the concept of catastrophes is similar as large scale disaster. Based on Chen et al. [13] with small adjustment, the characteristics of a large-scale disaster can be categorized as: Large scale impact, severe consequences, multi-agency involvement, time pressure, demand surge and resource shortage, great uncertainty, and infrastructure damage, as described in Table 2.

### 3.2. Challenges for Emergency Logistics

Chen et al. [13] studied the coordination support activities based on the challenges of disasters characteristics on emergency response. Based on the unique features of post-disaster humanitarian logistics, Holguin-Veras et al. [14] also analyzed the differences between commercial and humanitarian logistics and identified the research gaps along seven key components: the objectives being pursued, the origin of the commodity flows to be transported, knowledge of demand, the decision-making structure, periodicity and volume of logistic activities, and the state of the social networks and supporting systems. Following the same approach, we investigate the potential challenges for emergency logistics according to each emergency characteristic discussed in Section 3.1, with the results summarized in Table 3.

#### 3.2.1. Problem Scale and Complexity

Large-scale disasters may affect wide geographical areas and large populations with severe damage. Emergency logistics tasks therefore are very complex and complicated, involving overwhelming damage assessment and demand estimation, allocation of variety of resources, complicated resource distribution in short period of time, organizing rescue operation and mass evacuation, etc. Moreover, these tasks are interrelated to each other and cannot be solved individually without considering their mutual impact. There are also existing hard-to-measure factors like unanticipated surge of local demand, transportation infrastructure damage as well as possibility of secondary disaster occurrence, etc. These features making the problem structures in emergency logistics are inherently chaotic and complex. Some of them are intractable and even containing numerically unsolvable instances from mathematical point of view. Hence, these substantial complexities and obstacles pose a great challenge for emergency logistics [6].

#### 3.2.2. Different Objectives and Decision Criteria

Large-scale disasters may cause large casualties and severe property damages. In this context, the objectives and decision criteria for emergency logistics should focus on saving lives, alleviating human suffering and reducing property damages, rather than the traditional objective of reducing operating costs and increasing profit for business [15,16]. Moreover, the objectives or decision criteria can be different even conflict among parties involved. For instance, lifesaving may conflict with damage control. The objective achievements are often ambiguous and hard to measure [17]. For example, “Imagine an organization whose mission is to alleviate human suffering. How can you measure such an abstract notion? How can an organization meaningfully assess its direct contribution to such a broadly stated mission?” [18].

#### 3.2.3. Multiparty Collaboration Problem

Large-scale disasters require multiple agencies such as police, firefighters, medical teams, red-cross, volunteers, etc, working together to carry out emergency response tasks [19]. With different authorities, functionalities and professions, multiparty coordination could be very complex. Not only do these enormous players have different incentives and motivations, but also may exacerbate the competition for limited resources. Therefore, good collaboration is needed for information exchange, resource sharing, and job dispatching among different parties [20]. Otherwise, a lack of collaboration can lead to disaster propagation and even higher numbers of casualties [21].

#### 3.2.4. Critical Time Requirement and Real-Time Decision Making

Disasters usually happen suddenly and develop rapidly. Any delay of relief efforts may cause severe consequences and many failures. Hence, there is time pressure to make quick decisions and to provide quick responses [15,22,23]. Under the situation of strict time pressure, two challenges are imposed on emergency logistics: On one hand, we need to speed up the response operation such as quick transportation of humanitarian aid through better scheduling. Sometimes it is necessary to look for a quick feasible solution rather than an unrealistically sophisticated optimal solution, because time is critical for quick emergency response. On the other hand, we need to speed up the decision-making process in order to reduce unnecessary delay. Real-time information gathering and decision support therefore is very critical [10].

#### 3.2.5. Allocation of Scarce Resources

Large-scale disasters may create a sudden huge demand for emergency resources which greatly exceeds resource availability [24]. In this situation, it becomes imperative to allocate these scarce resources for different demand areas and ensure their availability to those that need resources the most [25]. Emergency resource allocation needs to consider many factors simultaneously, such as damage scenarios, number of casualties, priority of demand fulfillment, urgency level of needs, as well as delay consequence of humanitarian aids. However, how to set up allocation principles and measure resource allocation performance is a subject of much debate [26], since besides efficiency and effectiveness, it unavoidably involves questions of justice and fairness. With urgent needs and insufficient resources, what are the justice and fairness for resource allocation? There is still lacking of consensus on what they should be [27], because it is a most controversial topic regarding of ethical issues [28].

#### 3.2.6. Stochastic and Scenario-Based Modeling

Large catastrophic disasters usually have little precursory features before their occurrence, which make them highly uncertain and difficult to predict. In the large-scale emergency response practice, it is usually hard to predict the scope and progress of a disaster situation, to assess the damages and to estimate the resource requirement accurately [29]. To cope with these uncertainties, it is necessary to establish stochastic or scenario-based emergency logistics models [30,31,32,33].

#### 3.2.7. Logistics with Damaged Infrastructure

Large-scale disasters may cause extensive damage to communications, power supplies, and transportation infrastructures, and make them unavailable for emergency relief operations. For example, disrupted transportation facilities including ports, airports, roads and bridges may limit the humanitarian aids access to disaster attacked regions; destroyed communication infrastructures such as telephone and radio towers can hamper information collection and transmission of the catastrophe so as to slow down the responsiveness. Hence, these additional constraints need to be taken into consideration for emergency logistics operations [34].

## 4. Current Studies and Future Research Directions

Based on the challenges to emergency logistics discussed in Section 3.2, we further review the achievements and the gaps of current OR studies in responding to these challenges, and identify future research directions. The findings are summarized in Table 4, followed by a detailed discussion in each subsection.

### 4.1. Problem Scale and Complexity

Emergency logistics can be viewed as a very complex dynamic process which consists of many interdependent tasks with complex objectives and constraints. For example, after an occurrence of a large-scale disaster, the primary task is collecting and distributing emergency resources to the affected areas. However, several interdependent tasks emerge, such as who holds the emergency resource, where can one get the emergency resource, who delivers the emergency resource to the affected areas, when are transport vehicles available, etc. Hence, emergency logistics is a complicated problem since various decision problems must be considered simultaneously.

Currently, emergency logistics problems have been studied in four areas: demand assessment, resource allocation, resource distribution, and emergency evacuation. Few studies integrate two or more specific decision problems into one decision model.

Damage assessment and demand estimation refer to the assessment of the damage from disaster affected areas and the estimation of possible resource requirements in these areas. Problems studied include damage assessment, disaster area grouping, demand requirement forecasting, and demand priority ranking. Moltchanova et al. [35] developed a stochastic model to evaluate the economic losses and loss of life to assist efficient earthquake response. Chang et al. [32] grouped the affected regions based on their geographic distribution and distance. Other works related to area grouping method can refer to Gong and Batta [36] and Jotshi et al. [37]. Sheu [7] investigated time-varying relief demand forecasting, disaster area grouping and information uncertainty evaluation. The gap on this topic is a need for demand assessment models that can provide information in multiple dimensions at different levels of aggregation and keep updating in a dynamic environment.

Resource allocation refers to allocating limited resources to disaster affected areas with the guidance of allocation principle. Fiedrich et al. first pointed out the significance of optimal resource allocation to disaster affected areas during the initial search-and-rescue period after a large-scale earthquake happened. Sherali et al. [38] discussed the problem of allocating certainly available resources to mitigate risks that may arise after the occurrence of natural disaster. Gong and Batta [36] considered ambulance allocation problem in immediate disaster response operations. Felder and Brinkmann [15] addressed the emergency medical service allocation amongst urban and rural regions. Zhang et al. [39] took the possibility of secondary disaster into account in the multi-resource allocation model. Arora et al. [25] studied the antiviral allocation problem to cope with a large-scale pandemic flu. They discussed the tradeoff between maintaining local redundant capacity and relying on mutual aid of antiviral resource. In fact, resource allocation problem usually involves many different types of resources, with very different requirements, e.g., periodical need or one-time need. However, current researches seldom consider this difference in the decision models.

Resource distribution discusses how to deliver the various relief resources to affected areas efficiently. To some extent, distribution activities play a central role in disaster response operations. We notice that it is important to identify specific features in disaster response, such as infrastructure damage, as well as availability and compatibility of various delivery tools. Without these features, it will be hard to distinguish the emergency distribution models from traditional distribution problems, e.g., Tzeng et al. [40,41]. Most studies formulate emergency resource distribution problem as vehicle routing problem. For instance, Haghani et al. [42] proposed a simulation model to assist emergency medical vehicle dispatching and routing decision through updating real-time travel information; Shen et al. [43] addressed a stochastic emergency vehicle routing problem in response to a large-scale bioterrorism emergency; Lin et al. [44] investigated a specific vehicle routing problem through taking prioritizing item delivery into account, and formulated it as a multiobjective integer programming model. Some scholars have studied route selection problem such as Yuan and Wang [45]. They developed a multi-objective route selection model with consideration of the travel speed on each route affected by disasters. Some other scholars have investigated road capacities. For example, Barbarosoglu and Arda [30] incorporated the randomness of transportation capacity into their model; Jotshi et al. [37] considered the data fusion of road condition in their proposed emergency vehicles dispatching and routing model. Another studying viewpoint is to choose a proper traffic modal according to road conditions and road capacities, e.g., distribution via helicopter in Barbarosoglu et al. [46] and Özdamar [47], multimodal transport routing optimization in Özdamar et al. [6].

Emergency evacuation studies how to displace people from dangerous areas to safe places. In OR, the evacuation problem is mainly formulated as a network design and network flow control problem with the objective of improving the efficiency of emergency evacuation. The interested reader is referred to Abdelgawad and Abdulhai [48] and Hamacher and Tjandra [49] to get more detailed review. In the existing literatures, the necessity and importance of OR models have been established [48], in which the typical OR approaches contain multiobjective optimization model [50], static network flow model [51], dynamic network flow model [52,53,54,55,56,57], time-expanded network model [44,58], and fuzzy robust programming model [59]. Hamacher and Tjandra [49] provided a detailed classification on mathematical modeling of evacuation problems. From the mathematical solvable point of view, Kim et al. [60] discussed the design of heuristics to solve large scale evacuation network flow model. Liu et al. [61] presented an algorithm applicable to evacuation problem in a specific context after a flood disaster occurrence.

Besides focusing on individual emergency logistic decision problems, some researchers also made efforts to integrate different specific decision problems into one decision model, such as integration of resource allocation and emergency distribution [62], combine vehicle routing with supply allocation [63], joint optimization of distribution network repair with relief distribution scheduling [34,64,65], coordination model between emergency distribution and evacuation [54,55], a transshipment model linked distribution with inventory relocation model [66], a combined stochastic model for the storage and distribution of medical supplies [67], and an integrated model of facility location, emergency resource delivery, and vehicle routing [68].

In general, previous studies have mainly focused on decomposition-oriented methods, which deal with emergency logistics problems by simplifying the problems or decomposing large problems into multiple smaller problems. The advantage of this paradigm is to make the complicated problems more tractable and easy solvable, but the major disadvantage is that it omits the interrelationships amongst the emergency relief activities. On the other hand, although the idea of an integration-oriented method such as joint optimization model in the context of large-scale disasters has been raised recently, it is challenging to build integrated models that address the entire emergency logistics process for large scale problems. The integration may also be achieved through an emergency response coordination system in which different models for different problems can be connected to each other through a network of information flows. For instance, the output of demand assessment can be used as input of resource allocation and the output and the output of resource allocation can be the input of resource distribution etc.

### 4.2. Different Objectives and Decision Criteria

The objectives and decision criteria not only reflect the attitudes and principles of decision-makers towards decision problems, but also act as measurements to assess various schemes or schedules. Effective decision metrics can not only help practitioners make quick decisions and improve emergency responsiveness, but also can benefit in coordinating much interdependent tasks amongst various participators and smoothing the disaster relief operations. Owing to the central role of emergency logistics in relief operations, the decision objective and criteria is critical for responding to large-scale emergencies and control the consequences of disasters [22].

In existing literature, objectives commonly used in emergency logistics can be classified into three groups: improvement of distribution performance, assessment of demand fulfillment, and reduction in human deaths or improvement in human survivability. Moreover, it is also possible to combine objectives from different groups as multiple objectives.

The criteria for improving distribution performance include: minimizing the selection of shortest path [39,50,69], minimizing distribution time [34,37,40,44,47,53,57,67,60,70], minimizing the time span of task completion [34,36], minimizing evacuation time [59,71], minimizing delay time of distribution service [52,54,55], minimizing distribution cost [7,30,39,40,41,46,62,66,72,73,74,75], minimizing evacuation network construction cost [71], minimizing the vehicle utilization [76], maximizing the outgoing flow [58,77], maximizing vehicle tour duration [46], as well as minimizing the average travelled distance [78].

The criteria to assess demand fulfillment include minimizing unsatisfied demand [43,44,52,54,55,68,76,79,80], maximizing demand fill-up rate [7,40,65], and minimizing the difference of demand satisfaction rates between different areas [44].

The criteria of reducing human deaths or improving human survivability include minimizing the number of fatalities [81,82] or maximizing the expected number of saved people [83], maximizing weighted throughput of casualties [84], maximizing the human survival probability in ambulance service [23,85,86], and possible health outcomes (death, hospitalization, outpatient care) in evaluating different intervention policies for influenza pandemics [3].

Unlike commercial logistics taking minimizing economic cost as primary performance measurement, emergency logistics metrics is more complicated and need to consider much complex factors [87]. Some researchers attempted to develop a performance measurement framework for emergency logistics decision. For example, Huang et al. [63] used equity, efficiency and efficacy as important indicators to measure emergency relief operations, and investigated the balance among the three metrics; Felder and Brinkmann [15] investigated the trade-off of equity and efficiency in emergency medical service; Davidson [4] used appeal coverage, distribution time, efficiency, and assessment accuracy to measure the performance of relief logistics; and Balcik and Beamon [22] suggested to apply the performance measurement framework of commercial supply chain to humanitarian relief chain, and developed a framework consisting of resource, output, and flexibility metrics through extending the previous work in Beamon [88]. Some literatures specially discussed the balance amongst different objectives in their developed models, such as the tradeoff of efficiency and equal service time in Chiou and Lai [64]. Holguín-Verasa et al. [89] argued that welfare economic principles must be incorporated in post-disaster humanitarian logistic models to ensure delivery strategies that lead to the greatest good for the greatest number of people. They suggested the use of social costs—the summation of logistic and deprivation costs—as the preferred objective function for post-disaster humanitarian logistic models where the deprivation cost was the economic valuation of the human suffering associated with a lack of access to a good or service. Recently, Eisenhandler and Tzur [90] investigated the tradeoff between effectiveness and equity for food allocation among welfare agencies, and formulated it as a routing resource allocation problem.

In summary, most current research focuses on traditional logistics objectives (minimization of distribution time and distribution cost, and selection of shortest path). Typically, cost-based and time-based objective functions are often representative of current research efforts. Few studies have considered emergency related decision criteria such as minimizing the number of fatalities or maximizing demand fulfillment. However, the primary principle in emergency logistics is saving human lives and reducing property damage through various emergency relief activities. Thus, the decision objectives and criteria of future studies should be more directly linked to the end results such as life-saving, human suffering alleviation as well as damage reduction [91]. Moreover, although the performance measurement of emergency logistics has received greater attention by many academics [22], a uniform metric framework to guide emergency relief operation needs to be further investigated.

### 4.3. Multiparty Collaboration Problem

Coordination can be defined as the management of interdependencies between activities to achieve a goal [92]. In emergency response tasks are often complex, uncertain, and interdependent to each other and need to be carried out jointly by multiple agents [93].

Current OR has studied the coordination of multiple decision problems. Yi and Özdamar [55] and Yi and Kumar [54] investigated the decision coordination problem between emergency distribution and emergency evacuation. Chiou and Lai [64] and Yan and Shih [34] integrated road repairing scheduling with relief distribution to analyze the integrated schedules. Huang et al. [63] combined the vehicle routing with supply allocation, under the consideration of equitable service to all beneficiaries. Balcik et al. [62] developed a joint optimization model of resource allocation and emergency distribution. Moreover, Rottkemper et al. [66] investigated the integration of resource distribution and inventory relocation, and formulated it as an integrated transshipment model. Generally speaking, the integrated optimization and assessment method is a good way to coordinate different relief activities in emergency logistics schedules. The integration of relief activities can improve the effectiveness and efficiency of emergency relief operation and enhance the responsiveness to emergency events [94,95], whereas the development of a more complex emergency logistics model may increase the problem complexity and will put more stress on model solutions especially under time pressure of emergency relief decision making.

Also, we notice that there are some OR literatures outside emergency logistics domain have made contributions to the topic of collaboration, such as collaborative transportation planning [96], shipper collaboration model [97], A detailed literature review of collaborative transportation is provided by Agarwal et al. [98]. We believe the basic principle of collaboration is common in different areas, and the collaboration approach in transportation domain may be applied to collaborative emergency relief operations.

On the whole, the multiparty collaboration problem still remains at the top of the research agenda [21,99]. Most current literature investigates this topic from a single authority’s perspective, i.e., assume a single authority to deal with emergency response. Although some studies have considered the coordination of multiple decision problems such as the integration of resource allocation and distribution, they have not considered different objectives by different parties. In fact, emergency logistics almost always needs to simultaneously implement different sequential response tasks [100]. Hence, future studies in this field should focus on the investigation of the interdependency of tasks, resources, and workflows across and among different decision authorities, and possible modeling tools could learn from the knowledge in the fields of collaborative transportation planning and workflow technology. The need for multiparty coordination has also been emphasized by Altay and Green III [3,10], and Galindo and Batta [1]. As various groups involved in emergency response, organizational structures and relationships need to be built into multi-criteria, multi-objective decision making models.

### 4.4. Critical Time Requirement and Real-Time Decision Making

Time is life in emergency response. Any delay of decision and action may cause unnecessary casualty and human suffering that otherwise could be avoided [86,101]. The highest priority for emergency logistics is to save lives as soon as possible, and emergency response time has been identified as a critical indicator to measure the performance of emergency relief operation and survival possibility of injured people [23]. Hence, many countries have enacted the time threshold to respond in large disaster events. For instance, under the new Department of Homeland Security in the U.S., the Strategic National Stockpile (SNS) Program is required to maintain a stockpile of pharmaceutical agents, vaccines, medical supplies, and equipment to augment state and local resources during a large-scale disaster or bioterrorism event. Upon request, the SNS Program will deliver materials anywhere in the United States within 12 or fewer hours [102].

To deal with critical time requirement, current OR studies tend to focus on making the minimum distribution time as the decision objective, or setting time windows as a constraint in the mathematical model. Those studies related to minimizing distribution time have been analyzed in Section 4.2. A typical study is conducted by Gong and Batta [36], who studied the problem of initial allocation and subsequent reallocation of ambulance amongst casualty clusters in consideration of round-trip service time. They developed a continuous function to depict the casualty growth in a cluster, and combined it with the criterion of minimax completion time that ambulances need to serve the casualty cluster. For studies of the time-window setting, Haghani and Oh [41] incorporated a time-window constraint into a time-space-based distribution network; Shen et al. [43] investigated an emergency vehicle routing problem in consideration of the time window constraint; and in Lin et al. [44], they took account of the soft time windows in their developed emergency relief planning model.

For quick decision making, some papers have investigated the problem of real-time decision making through continuously updating information used in decision models. Thus, this requires the rapid data gathering and information processing, and continuous adjustment with the changes of disaster situations [103]. In current literatures, the widely used method for real-time information processing is data fusion, which is a process that refines its estimations and assessments of decision parameters continuously. This includes Sheu [104] who adopted data fusion methods to forecast relief demand in multiple areas, so as to support the emergency distribution, and Jotshi et al. [37] who estimated the number of casualties and road conditions in a post-disaster environment by using the data fusion method. In addition, combine efficient optimization technology with real-time decision support system is another interesting area of research to assist emergency response [42,105]. For example, Horner and Downs [106] and Chang et al. [32] incorporated the geographic information system (GIS) into the emergency rescue planning model.

Some OR studies attempted to analyze the critical time requirement for life saving perspective and explored the quantitative relationship between critical response time and human being survivability. Felder and Brinkmann [15] pointed out that the response time can crucially determine the quantity and quality of life saving in an emergency event. Erkut et al. [23] and Knight et al. [86] investigated the patient survival possibility in the context of ambulance location models, and demonstrated that the probability of patient survival was a function of response time. Moreover, McLay and Mayorga [85] also discussed the performance evaluation of response time thresholds in terms of resulting patient survival rates.

Although current literatures have taken time into consideration, most of them treated time as an objective or a constraint from the traditional logistics perspective, and did not reflect the time pressure feature in the aftermath of a large-scale disaster. Thus, quantitative metrics linking human survivability with response time is very much needed. This issue has been recognized and investigated by some scholars, but their studying efforts are all based on the statistical results from medical care field [23,86], and may inadequate in the case of general emergency logistics. We also need to make more efforts to address the issues of decision support user interface for real-time applications of decision models [10]. Finally, exploring time-varying process between disaster scenarios and time criticality is rare in current literatures, and need to be further investigated.

### 4.5. Allocation of Scarce Resources

During a disaster, resources need to be allocated to the affected people and places for effective rescue operation. A general issue in this problem domain is how to allocate emergency resources in order to relieve the consequences caused by a disaster [15,38]. In a large-scale disaster situation, resource allocation decisions are often affected by sudden demand surging and serious shortage of available resources. In the context of insufficient emergency resources, different criteria can result in different resource allocation schemes. Usually, priority setting is a key determining factor to allocate scarce emergency [107].

To deal with resource shortage, we borrow the worth-oriented paradigm from social choice theory [108] and welfare economics [109]. According to their opinions, the competing ethical principles for scarce resource rationing are utilitarianism and egalitarianism. Utilitarianism focuses on maximizing total social worth or maximizing the greatest value of goods for the greatest number of people, which can also be interpreted as the most lives saved in the context of emergency response. The utilitarian principle is to ration the scarce emergency resources and determine the satisfaction of demand requirements through priority setting [110]. For example, the priority in emergency relief operations may be related to which affected region should be satisfied first, triage protocols in places [24,111], etc. However, this allocation principle may cause inequality amongst disaster victims at different affected regions. Caro et al. [28] argued that utilitarian efficiency should be tempered by the principle of equality in making decisions about providing life saving interventions and palliation. Although utilitarian efficiency is important, egalitarian criteria (i.e., equality or fairness) are also the key modifying ethical principle. Bertsimas et al. [112] even argued that fairness should be obtained at the expense of efficiency sacrifice in resource allocation. They also analyzed different price of fairness such as proportional fairness and max-min fairness in their discussion. According to Winslow [110], egalitarianism was based on equality of opportunity and fairness of demand satisfaction. In the immediate emergency relief operations, equality or fairness not only means the injuries have equal rights to have their needs met [28], but also refers to the variance in arriving times of resources should be as small as possible [63,91].

The criteria for allocation decisions in the current literature can be grouped as two streams: one only focused on the utilitarian principle, and another considers both of utilitarian and egalitarian principles. For the stream of utilitarian principle, different utilitarian criteria have been embedded into the developed models, such as cost-effectiveness analysis [113], cost-benefit-based methods [25], deterministic priority setting [36,38,39,53], triage management policy [24,111], urgency level of disaster affected regions [7,68], injury classes ranking [81], humanitarian aids criticalities [62]. Although these criteria seem distinctive from each other and a bit dazzling, the essential of them is the same. That is, allocate the scarce resource or emergency service through prioritizing the affected regions or injuries. For the stream of following both utilitarian and egalitarian principles, Felder and Brinkmann [15] combined the efficiency (i.e., maximizing the total number of survivors) and equality (i.e., equal access to emergency medical service) in their developed emergency medical service allocation model, and find that the two objectives can lead to different deployment patterns. Jacobson et al. investigated the tradeoffs among demand urgency, rescue rewards and service times in allocating emergency resource to multi-categorization casualties, and formulated this problem as a priority assignment policies optimization model. De la Torre et al. [91] reviewed the allocation policies from the perspective of practitioners and academics. Besides that, there are three articles that are closely related to the latter stream, namely, Ferrer et al. [114], Huang et al. [63] and Mete and Zabinsky. Both of them investigated the metrics of equality and efficiency for emergency distribution.

Generally, resource allocation criteria in current studies are focusing on utilitarian principle, and only few studies have considered the balance between efficiency and equality in their developed models. Current studies often consider the allocation of single type of resources. But in practice resource allocation usually need to deal with multiple types of resources that related to each other with different allocation principle. Current literatures usually rank affected regions with deterministic priorities and seldom consider the ranking priority changed over time. In fact, resource allocation is related to resource distribution capacity for delivery and cannot be decided separately in a sequential order, thus resource allocation needs to be adjusted frequently in order to respond to the changing situation such as surge of casualty. Hence, joint resource allocation and distribution needs to be studied.

Future studies in this field can be presented from two aspects. On one hand, we need to develop more dynamical priority metrics to allocate emergency relief resources. From the utilitarian perspective, one can develop dynamic efficiency criteria in emergency resources allocation by introducing the law of diminishing returns, in which plenty of works in the economics area can be referred. From the egalitarian perspective, one can discuss the nonlinear consequence of humanitarian aids delay or of injuries’ waiting cost. And its model development can be learned from the problem formulation of delay and tardiness in the machine or job shop scheduling field. On the other hand, combine priority setting and demand fulfillment as the criteria for resource allocation, and achieve a balance between priority and equality. Although current studies have considered prioritization in resource allocation decisions, how to set priorities is still an open problem for emergency relief actors [115], and even need interdisciplinary studies devoted into this field [116,117]. In the context of demand requirement greatly exceeds resource supplies, emergency response should consider both resource shortages, human life equality, and need urgencies of disaster victims. Thus, future research in scarce resource allocation needs to take resource utilization efficiency, emergency relief equality and human life time into account simultaneously [28,107]. Some other studies on efficiency versus equality (or fairness) will be helpful to understand and formulate the tradeoff issues of allocating scarce relief resources, and interested readers can refer to these studying efforts in Hooker and Williams [118], Mandell [119], Golany and Tamir [120], Gloverand Ball [121], Bertsimas et al. [122], Jacobson et al. [107].

In general, there are often resource shortages during emergency response. However, material convergence may also happen when hundreds of thousands of donors (governments, communities or individuals) sent massive amounts of supplies and equipment during a disaster. Although it brings in much-needed supplies, a significant portion of useless unsolicited donations creates major complications for the disaster response [14]. There is an urgent need to study the dynamics of the material convergence and the ways to control and reduce the negative impact of non- and low-priority material flows [14].

### 4.6. Stochastic and Scenario-Based Modeling

There is great uncertainty in the context of large-scale disasters. The uncertainty exists in many aspects such as demand requirements, supplies availability, road conditions and damage levels, etc. There are also great uncertainties associated with human behavior in disaster situations [11]. The difficulty of information gathering and the damaged communication infrastructure during disaster make the degree of uncertainty even worse.

Klibi et al. [123] defined uncertainty as the inability to determine the true state of the future business environment which may be partially known or completely unknown. They distinguished three types of uncertainties: randomness, hazard, and deep uncertainty. Randomness can be characterized by random variables related to business-as-usual operations. Hazard is characterized by low probability unusual situations with a high impact, and deep uncertainty is characterized by the lack of any information to access the probability of plausible future events. Therefore, the methods adopted to handle these different kinds of uncertainties should be adapted with their intrinsic attributes [124]. For the randomness problems, the common way is to model the uncertainty as random variables, and make “robust” decisions prior to uncertainty being realized, as in stochastic programming or robust optimization approaches. For hazards, it may be very difficult to obtain sufficient data to assess objective probabilities and subjective probabilities must often be used. While for the deep uncertainty problems, the tackling methods are to make prompt response after uncertainty became certain. Herein, we also follow their opinions in our discussion, because natural disasters especially the catastrophic emergencies are quite difficult to obtain sufficient information to predict their occurrence [125].

Large-scale disasters usually have the intrinsic feature of deep uncertainties, and pose many hard-to-measure factors and stringent constraints on immediate emergency logistics decision making, but it does not mean those uncertainties cannot be predicted completely in practical emergency response operations. Indeed, some uncertain decision variables can be estimated approximately. For example, the uncertainty of supply amount can be handled by summing up the total resource delivered to the affected region until the decision making epoch. The uncertainty of demand requirement also can be estimated by the demographic information of the disaster attacked regions. Therefore, in emergency logistics for this kind of uncertainties, they can be characterized from previous experience or forecast. However, some other uncertainties have no laws and are difficult to predict or estimate. For example, a road is damaged after earthquake. We know the transportation on the road will be delayed, but it is very hard to use a random variable to characterize the randomness very well, since the road conditions dynamically varied with the possible earthquake aftershocks and emergency repairs. In this context, responsive decisions methods after the random events happen are more useful and practical than robust decisions before the random events happen. Therefore, in OR research works the modeling methods of disaster uncertainty should be in accordance with the problem characteristics, otherwise the conclusions and results may have not application values in practice [1].

At present, the common methodology used in OR community to deal with uncertainty include stochastic programming [30], scenario-based modeling [32], robust optimization [76,126], rolling horizon approach [66,127], fuzzy programming [59], as well as simulation approach [37], among them stochastic and scenario-based methods are more popular in model development. Stochastic programming has good ability to cope with uncertainties of disaster development through incorporating probabilistic scenarios [128]. For scenario-based approach, the advantage is that it can make a complicated problem more tractable [129], while the disadvantage is that it is difficult to deal with infinite number of disaster scenarios [123]. For more information on optimization under uncertainty, please refer to a survey paper by Sahinidis [130].

In the field of emergency logistics, the majority of current studies focus on the deterministic optimization models with the assumption of known data for given situations, and few works have investigated the stochastic models. Barbarosoglu and Arda [30] investigated the uncertainties of demand, supply and transportation capacity in emergency resource distribution scheduling, and developed a scenario-based two-stage stochastic programming to robustly disclose these uncertainties along with the progress of the emergency response. Reference [59] proposed a robust fuzzy programming to formulate the evacuation problem under uncertainty, and used a fuzzy lower and upper bound approach to handle the uncertainties of vehicle number, vehicle capacity, travel time, and number of waited trapped people, etc. Najafia et al. [76] developed a multi-objective robust optimization model for both of the disaster relief distribution and injured people evacuation in the aftermath of an earthquake happened. Shen et al. [43] considered the uncertain travel time and demand in the context of responding a large-scale bioterrorism emergency, and developed a two-stage stochastic vehicle routing model. In the first planning stage, they modeled the uncertainties as chance constraints, and coped with the chance constraints through revealing demand level and travel time in the second operational stage. Beraldi et al. [131] also used the chance constraint technique to hedge the emergency service reliability uncertainty in emergency service site locations and emergency vehicle assignments, and formulated the problem as a stochastic programming model. Mete and Zabinsky investigated the medical supply location and distribution to prepare and respond uncertain disaster scenarios, and formulated this problem as a two-stage scenario-based stochastic programming model. In their study, they adopted the scenario-based method to depict the plausible disaster scenarios and their emerged probabilities, and they then considered warehouse selection and inventory level decisions in the first preparedness stage, and medical resource distribution and demand satisfaction decisions in the second response state. The disaster-scenario-based modeling were also adopted by Chang et al. [32] and Li et al. [33], in which they respectively developed a flood-scenario-based mix integer programming model to assist the emergency preparations for floods and a hurricane-scenario-based bi-level programming model to prepare the attacks by possible hurricane events. Noyan et al. [132] investigated a post-disaster last mile distribution problem under uncertainty, and formulated it as a two-stage stochastic programming model. Balcik et al. [62] used a rolling horizon approach to real-time update the observed information and handle the uncertainties of resource supply and demand requirement. The information updating approach to handling uncertainties is also adopted by Chen and Miller-Hooks [83]. They studied a dynamical search and rescue team deployment problem over decision horizons. They considered the uncertainties of demand, service time and travel time, and formulated them as a multistage stochastic programming, in which they handled uncertainties through continuously updating the observed post-disaster information at each decision stage, so as to improve the robustness of emergency decision. In our understanding, the idea that prompt response after uncertainty became certain could be grouped into the umbrella of rolling horizon approach. Besides these formulations, another useful method for dealing with uncertainties is simulation, which was introduced in the emergency vehicle dispatching and routing [37], emergency evacuation [51] and resource allocation [105].

In conclusion, current studies in this problem domain have developed some robust stochastic models to hedge the uncertain conditions in the context of immediate emergency response, but they have not carefully distinguished the differences among those uncertainties emerged in real emergency relief operations, and the inherent complexity of uncertainty therefore is not adequately captured.

Further research on the uncertainty problem can be addressed from two aspects. On one hand, combine the disaster scenario generation with traditional optimization models. The big challenge might existed is that the plausible disaster scenario maybe infinite, especially in the context of high uncertainty and rapid disaster evolution. Under this circumstance, the appropriate alternative is adopting Monte Carlo method to generate the number of possible disaster scenarios [124,133]. On the other hand, deal with the difficulties in various kinds of uncertainties in an emergency situation, especially for those unprecedented emergency situations that no probability distributions are available. The major challenge of further studies on this aspect may come from modeling difficulty by using traditional OR methodologies, but catastrophe modeling techniques may provide a way to unlock this difficulty [125]. Catastrophe modeling is used to assess catastrophic risk and to improve risk management strategies. The modeling of catastrophe risk is a complex process that depends on scientific knowledge and subjective and objective inputs related to natural hazard such as hurricanes, floods, and earthquakes. Computer simulation is often used to estimate the risk of catastrophic events and assess the impact of possible actions. Catastrophe modeling tools are provided by software companies such as AIR Worldwide (www.air-worldwide.com), EQECAT (www.eqecat.com), and Risk Management Solutions (www.rms.com), and are widely used for risk analysis by insurance companies but it can have potential to be used in emergency logistics operations. Geographic Information Systems (GIS) with satellite image and remote sensor systems are also very useful for emergence management. GIS is capable to process, analyze and display spatial information for emergency response and have been used by emergency managers as a valuable decision support tool in emergency response in various kinds of disasters, including floods, bushfires, droughts, hailstorms, tsunamis, hurricanes, landslides, and earthquakes [134,135,136].

### 4.7. Logistics with Damaged Infrastructure

Emergency logistics often needs to deal with last mile distribution which refers to delivery of relief supplies from local distribution centers to beneficiaries affected by disasters [62]. In practice, last-mile emergency logistics always plays a central role among all emergency relief actives [62], and its successful operations can improve the robustness and flexibility to respond major disasters. During a disaster such as an earthquake, transportation infrastructures were often damaged, resulting in many constraints imposed on last-mile emergency logistics and affecting feasibilities for emergency relief operations.

Two approaches are used to handle the emergency logistics with damaged transportation infrastructure. One approach is to combine emergency logistics scheduling with damaged infrastructure repairing planning [34,64,65]. Another approach is to improve the robustness and flexibility for emergency relief operations. That is, consider possible uncertainties and additional constraints when making emergency logistics planning. Various circumstances from different perspectives have to be taken for consideration. Among them, capacity constraint is the most popular factor that to be considered in current studies, such as adding traffic capacity constraints into the distribution network [41], road capacity in emergency evacuation [53,59]. In addition, some other factors are also studied, such as road congestions, road complexities, etc. For example, Jotshi et al. [37] considered the road congestions in their emergency logistics network, and Han et al. [51] developed a one-destination evacuation model to avoid the road congestion or blockage; Yuan and Wang [45] investigated the shortest emergency distribution problem with the consideration of road uncertainty caused by disaster damage. Besides that, there are some other research streams related to damaged infrastructures, which include infrastructure vulnerability analysis [137], emergency recovery management [138].

In summary, considering capacity constraint is the most common way used in emergency logistics models to cope with the impact of damaged infrastructure, which is usually regarded as a lower bound or an upper bound with given disaster scenario. However, how to determine the lower and upper limit is still a difficult issue, since the condition of infrastructure damage is dynamic and varied with the situation changes in large-scale disaster. Further research on this problem can be focused on alternative solutions, such as investigating the combinatorial choice of multi-mode transportation routing to cope with infrastructure damage/availability. On the other hand, improve the resilience capability of emergency logistics network to resist the disaster attack is another research direction. Here resilience refers to the robustness and flexibility against emergency events and quick recovery capability from disruptions [139], which has been paid increasing attention in the fields of transportation planning [140] and supply chain design [141]. Hence, building the resilience into emergency logistics network can not only improve its reliability under a deep uncertain environment, but also can reduce the restoration time from emergency event strikes [142].

## 5. Conclusions

In this paper, we used the problem identification and solving approach to review existing emergency logistic literature. The main contributions and novel studies are summarized as follows. Firstly, we identified the key characteristics of large-scale disasters and assessed their challenges to emergency logistics. Secondly, we then analyzed and summarized the current literature that deals with these challenges. Finally, we discussed existing gaps in the current research and indicated future research directions.

Our review approach is significantly different from simple summery or classification based on the years of publication, the algorithm used, the application area, the types of logistic problems, or the phases of emergency management. The main contribution of our work is to provide a new perspective to re-exam emergency logistic research. We cannot simply apply our knowledge of business logistics to emergency logistics without deep understanding of the unique characteristics and challenges of large-scale disasters. When we have understood these challenges, we can thus identify what we have accomplished and what are the gaps and further research directions.

To deal with the challenges of large-scale disasters, we need to change many basic assumptions we usually use in traditional business logistics. For instance, we need to shift our focus from problem decomposition to multi-task integration, from time for operation efficiency to time for life saving, from single decision-making authority to multiparty authority, from unlimited resource availability to serious resource shortage, from uncertainty with randomness to deep uncertainty without previous knowledge, from perfect transportation infrastructure to damaged infrastructure. We believe that under those assumption changes we can significantly enrich OR in emergency logistics.

A limitation of our work lies in that we only concern the phrase of immediate emergency response and survey current OR literatures contributed to this phrase. Actually, the framework of emergency logistics is broad, which could extend into preparedness operations before the occurrence of disasters as well post-disaster restoration activities. Another limitation is that we limit the scope of our review in the academic community, and haven’t included the works on disaster response from the practice viewpoint by some key agencies like Red Cross, WHO, UN, etc. Hence, these limitations provide a direction to enrich and improve our work in future research.

## Figures and Tables

**Table 1:** Summary of review papers on emergency logistics.

Review Papers	Contributions
Green and Kolesar [8]	Analyzed previous OR papers on urban emergency services and regular emergencies.
Wright et al. [9]	Extended the literature scope into homeland security such as traffic and cyber space safety in routine emergency management, but not in the context of large-scale disasters.
Altay and Green III [3]	Provided disaster operations management literature classification in six dimensions. Highlighted the gaps in multi-agency coordination, soft OR, the use of sensing technology, recovery planning, business continuity, and management engineering.
Simpson and Hancock [10]	Discussed the literature on both urban emergency service systems in early days and of large-scale disasters in 21st century. Identified a detailed literature citation network. Suggested four areas of research opportunity.
Caunhye et al. [11]	Reviewed optimization models in emergency logistics in the pre-disaster operations phase and post-disaster operations phase. Analyzed the literature contents in detail through the perspectives of OR models, decisions, objectives, and constraints.
Galindo and Batta [1]	Extended Altay and Green’s survey on disaster operations management and added analysis on most common assumptions in the field. Suggested further research areas.

**Table 2:** Key characteristics of large-scale disasters.

Emergency Characteristic	Description
Large scale impact	May affect wide geographical areas and large groups of population
Severe consequences	May cause huge number of casualties and severe property damages
Multi-agency involvement	May involve multiple parties such as rescue teams, volunteers, and international support teams
Time pressure and emergency	Time is critical for life saving, and there is time pressure for quick decision making and action
Demand surge and resource shortage	Huge demand surge with severe resource shortages
Great uncertainty	Great uncertainty caused by the nature of the disaster which is often unpredicted and unprecedented
Infrastructure damage	Infrastructure is often damaged, becoming inaccessible or unusable

**Table 3:** Challenges for emergency logistics.

Emergency Characteristic	Challenge for Emergency Logistics
Large-scale impact	Problem scale and complexity
Severe consequences	Different objectives and decision criteria
Multi-agency involvement	Multiparty collaboration problem
Time pressure and urgency	Critical time requirement and real-time decision making
Demand surge and resource shortage	Allocation of scarce resource
Great uncertainty	Stochastic and scenario based modeling
Infrastructure damage	Logistics with damaged infrastructure

**Table 4:** Current studies and future research directions in emergency logistics.

Challenges	Current Studies & Limitations	Future Research Directions
Problem scale and complexity	▪ Most studies focused on decomposition to a specific or simplified decision problem, such as demand assessment, resource allocation, emergency distribution, and emergency evacuation; ▪ Few studies considered an integrated model.	▪ Develop integrated models that address the entire emergency logistics process for large scale problems.
Different objectives and decision criteria	▪ Most studies focused on traditional logistics objectives (minimization of distribution time, cost and shortest path selection, etc); ▪ Few studies considered emergency specific decision criteria such as minimizing number of fatalities, maximizing demand fulfillment, and minimizing unsatisfied demand.	▪ Make objectives more directly link to end results such as life- saving and damage reduction; ▪ Develop a uniform metric framework to guide emergency relief operation.
Multiparty collaboration problem	▪ Most studies assumed a single authority to deal with emergency response; ▪ Some studies considered the coordination of multiple decision problems such as the integration of resource allocation and distribution, but did not consider different objectives by different parties.	▪ Investigate task, resource, and workflow interdependency across different stakeholders.
Critical time requirement and real-time decision making	▪ Current studies tended to focus on minimizing distribution time and setting time windows as a constraint; ▪ Some papers enabled real-time decision making through continuously updating the information used in decision models; ▪ Few researches linked human survival possibility with emergency response time; ▪ Lack of study on real-time decision-making implementation issues.	▪ Explore the measurement of critical time requirement; ▪ Develop more adequate quantitative metrics linking life- saving with response time; ▪ Make more efforts to address the decision support user interface issue for real-time application of decision models; ▪ Explore dynamic relationship between disaster scenarios and time criticality.
Allocation of scarce resources	▪ Resource allocation was usually based on a given priority, and seldom consider the priority changed over time; ▪ Lack of consideration on multi-type resource allocation; ▪ Few studies considered the balance between efficiency and equality.	▪ Develop more dynamical priority metrics to allocate relief resources; ▪ Combine priority setting and demand fulfillment as the criteria for resource allocation; ▪ Achieve a balance between priority and equality.
Stochastic and scenario-based modeling	▪ Most papers investigated deterministic models, with the assumption that data were known for the given situation; ▪ Few studies developed stochastic, fuzzy, and simulation models to tackle the uncertainties in disaster relief operations.	▪ Combine scenario technique with optimization model; ▪ Deal with the difficulties of unprecedented emergency situations (no probability distributions are available).
Logistics with damaged infrastructure	▪ The repair of damaged roads was incorporated into the distribution model; ▪ Traffic capacity constraints were added into distribution networks; ▪ Traffic capacity constraint was treated static, not dynamic during emergency response.	▪ Use combinatorial choice of multi-mode transportation to cope with infrastructure damage/availability; ▪ Improve resilience capability of emergency logistics network.
